# Anesthesia and surgery induce age-dependent changes in behaviors and microbiota

**DOI:** 10.18632/aging.102736

**Published:** 2020-01-24

**Authors:** Ning Liufu, Ling Liu, Shiqian Shen, Zengliang Jiang, Yuanlin Dong, Yanyan Wang, Deborah Culley, Gregory Crosby, Minghui Cao, Yuan Shen, Edward Marcantonio, Zhongcong Xie, Yiying Zhang

**Affiliations:** 1 Department of Anesthesiology, Sun Yat-Sen Memorial Hospital, Sun Yat-Sen University, Guangzhou, Guangdong Province 510120, P. R. China; 2 Geriatric Anesthesia Research Unit, Department of Anesthesia, Critical Care and Pain Medicine, Massachusetts General Hospital and Harvard Medical School, Boston, MA 02114, USA; 3 Department of Anesthesia, Critical Care and Pain Medicine, Massachusetts General Hospital and Harvard Medical School, Boston, MA 02114, USA; 4 College of Biosystems Engineering and Food Science, Zhejiang University, Hangzhou 310058, P. R. China; 5 Department of Anesthesia, Shanghai 10th People’s Hospital, Anesthesia and Brain Research Institute, Tongji University, Shanghai 200072, P. R. China; 6 Department of Anesthesiology, Perioperative and Pain Medicine, Brigham and Women’s Hospital and Harvard Medical School, Boston, MA 02115, USA; 7 Department of Psychiatry, Shanghai 10th People’s Hospital, Anesthesia and Brain Research Institute, Tongji University, Shanghai 200072, P. R. China; 8 Divisions of General Medicine and Primary Care and Gerontology, Department of Medicine, Beth Israel Deaconess Medical Center and Harvard Medical School, Boston, MA 02215, USA

**Keywords:** age, gut microbiota, mitochondria, neuroinflammation, postoperative delirium-like behavior

## Abstract

The neuropathogenesis of postoperative delirium remains mostly unknown. The gut microbiota is implicated in the pathogenesis of neurological disorders. We, therefore, set out to determine whether anesthesia/surgery causes age-dependent gut microbiota dysbiosis, changes in brain IL-6 level and mitochondrial function, leading to postoperative delirium-like behavior in mice. Female 9 or 18 months old mice received abdominal surgery under 1.4% isoflurane for two hours. The postoperative delirium-like behavior, gut microbiota, levels of brain IL-6, PSD-95 and synaptophysin, and mitochondrial function were determined by a battery of behavioral tests, 16s rRNA sequencing, ELISA, Western blot and Seahorse XFp Extracellular Flux Analyzer. Intragastric administration of Lactobacillus (10 days) and probiotic (20 days) were used to mitigate the anesthesia/surgery-induced changes. Anesthesia/surgery caused different alterations in gut microbiota, including change rate of reduction in the levels of gut lactobacillus, between the 18 and 9 months old mice. The anesthesia/surgery induced greater postoperative delirium-like behavior, increased brain IL-6 levels, decreased PSD-95 and synaptophysin levels, and mitochondrial dysfunction in 18 than 9 months old mice. Treatments with Lactobacillus and probiotic mitigated the anesthesia/surgery-induced changes. These data suggest that microbiota dysbiosis may contribute to neuropathogenesis of postoperative delirium and treatment with Lactobacillus or a probiotic could mitigate postoperative delirium.

## INTRODUCTION

Delirium, a syndrome of confusion and inattention, is the most common postoperative complication in senior patients and is associated with accelerated cognitive decline and Alzheimer’s disease and related dementias (ADRD) [1–4]. However, the neuropathogenesis of postoperative delirium remains mostly unknown [5], the mechanistic research in the area is limited, and no effective medication currently exists to prevent or treat postoperative delirium. Senior patients are more likely than younger ones to develop postoperative delirium ([6], reviewed in [7]), but the reason for this age-dependency is not clear.

The gut microbiota contains up to 95% of the entire human microbiota [8]. Gut microbiota dysbiosis, a condition of microbiota imbalance or maladaptation inside the gut [9], is an unhealthy microbiota relative to the communities observed in healthy persons, which could lead to altered immune functions and increased risk of disease [10].

Aging is associated with changes in the gut microbiota [11–13] and microbiota dysbiosis has been suggested to be associated with disorders of the immune, endocrine, and central nervous systems, including with ADRD [14–16]. Specifically, Alzheimer’s disease (AD) patients have decreased microbial diversity and the changes in gut microbiota of AD patients are characterized by decreased Firmicutes, increased Bacteroidetes, and decreased Bifidobacterium compared to cognitively normal controls [17]. AD transgenic mice have a significant shift of the gut microbiota compared to wild-type mice [18] and treatment of these animals with antibiotics on postnatal day 14 to 21 leads to long-term changes in the gut microbiota and decreased level of Aβ in the brain [19]. Further, an imbalance in the gut microbiota in AD transgenic mice promotes infiltration of peripheral immune cells into the brain, enhances microglial activation, and contributes to cognitive impairment and brain levels of Aβ increase [20].

Neuroinflammation may contribute to the neuropathogenesis of postoperative delirium. [5, 21, 22] However, while all patients may have surgery-induced increases in blood levels of pro-inflammatory cytokines (e.g., IL-6), which can enter the brain through an impaired blood-brain barrier and induce neuroinflammation [23–25], not all patients develop postoperative delirium. Thus, neuroinflammation alone (single-factor model of postoperative delirium) is not sufficient to cause postoperative delirium. Instead, it is likely that an additional vulnerability or predisposing factor for neuroinflammation and delirium is required. We hypothesized that one of these changes is gut microbiota dysbiosis associated with aging.

Specifically, gut microbiota dysbiosis may open the blood-brain barrier (BBB) [26] via cytokine (e.g., IL-6), immunological, hormonal, and neuronal signals ([27, 28], reviewed in [8]), which promotes cognitive impairment and AD neuropathogenesis by allowing passage of amyloid, lipopolysaccharide, and other neuroactive molecules into the brain [8, 17, 26, 29, 30]. Antibiotic cefazolin changes the gut microbiota and attenuates postoperative neurocognitive disorder in mice [31]. Previous studies showed that the adult mice (8 weeks old) with the postoperative delirium-like behavior and the adult mice without the postoperative delirium-like behavior had different gut microbiota composition [32]; and anesthesia/surgery caused changes in both gut microbiota and cognitive impairment in 18 months old mice [33]. However, these studies did not compare the anesthesia/surgery-induced changes in gut microbiota and delirium-like behaviors between the adult mice and the older mice, thus, the role of the age-dependent gut microbiota dysbiosis as the vulnerability to the postoperative delirium-like behavior remains largely to be investigated. Moreover, these previous studies did not determine the potential age-dependent changes in brain IL-6 levels and mitochondrial function in the mice.

Probiotics are live microorganisms that are intended to have health benefits when consumed. Lactobacillus and Bifidobacterium are among the most common probiotics. Probiotics are used for the treatment of diseases in many cases of clinic research [34–36].

Therefore, the objective of the current study was to test the hypothesis that anesthesia/surgery causes age-dependent changes in the gut microbiota and delirium-like behavior in mice that is attenuated by preoperative prophylaxis with Lactobacillus or a probiotic. To test this hypothesis, we used 9 and 18 months old mice, employed a battery of natural and learned behavioral tests to determine the delirium-like behavior [37], assessed the gut microbiota before and after the anesthesia/surgery by gene pyrosequencing of 16S rRNA, and measured levels of brain IL-6, synaptic marker and mitochondrial function as measures of neuroinflammation and cerebral dysfunction. We found that the anesthesia/surgery was able to induce age-dependent postoperative delirium-like behavior, changes in gut microbiota, levels of brain IL-6 and synaptic marker, and mitochondrial dysfunction in the mice.

## RESULTS

### Anesthesia/surgery induced age-dependent changes in behavior in mice

**Figure 1:**
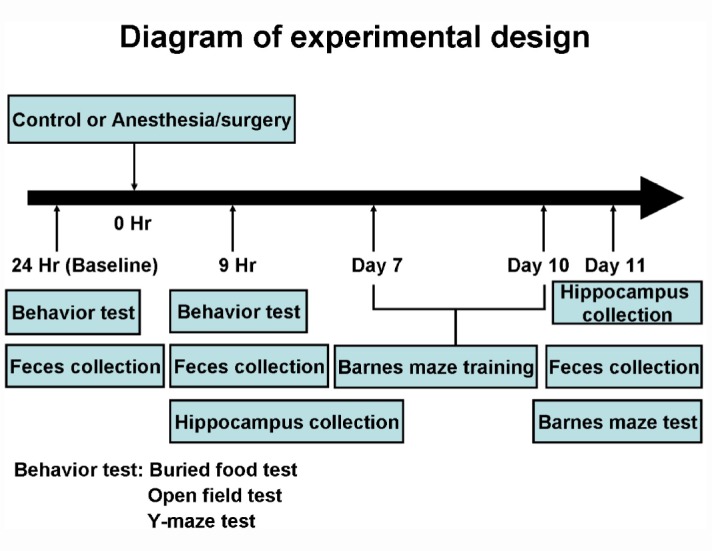
**Experimental design.** At the 24 hours before the control condition or anesthesia/surgery, the mice had the behavioral tests. At the 9 hours after the control condition or the anesthesia/surgery, the mice repeated the same behavioral test. The same mice then had Barnes maze training from day 7 to day 10, and Barnes maze testing on day 11 after the control condition or the anesthesia/surgery. The feces of the mice were collected at 24 hours before, 9 hours after and 11 days after the control condition or anesthesia/surgery before the behavioral tests. A different group of mice was used for the collection of the hippocampus at the 9 hours or 11 days after the control condition or the anesthesia/surgery.

We set out to determine the potential age-dependent changes in behaviors, gut microbiota, brain IL-6 levels, levels of brain synaptic marker, and mitochondrial function in 9 and 18 months old mice. The experimental design was demonstrated in Figure 1. There were no significant differences in blood pressure and heart rate (Supplementary Table 1), or the values of blood gas and electrolyte (Supplementary Table 2) between the 9 or 18 months old mice in the control condition and the 9 or 18 months old mice that received the anesthesia/surgery. These data showed that the anesthesia/surgery in the current studies did not significantly disturb physiology status of the mice.

**Figure 2:**
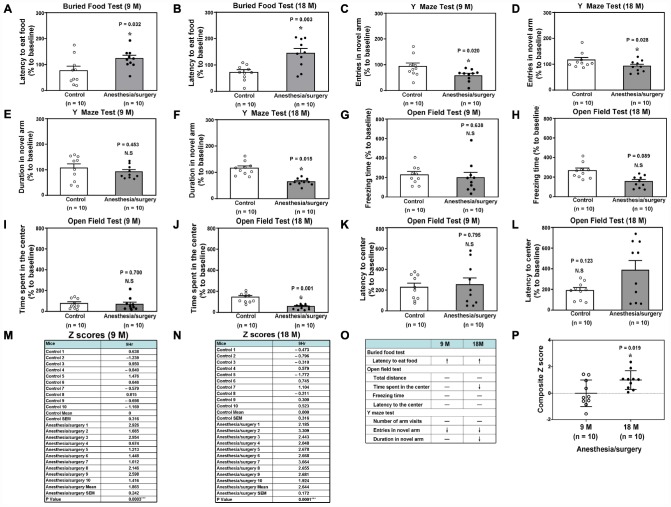
**Anesthesia/surgery induces age-dependent postoperative delirium-like behaviors in mice.** Buried food test in the 9 (**A**) or 18 (**B**) months old mice. Y maze test (Entries in novel arm) in the 9 (**C**) or 18 (**D**) months old mice. Y maze test (Duration in novel arm) in the 9 (**E**) or 18 (**F**) months old mice. Open field test (Freezing time) in the 9 (**G**) or 18 (**H**) months old mice. (**I**) Open field test (Time spent in the center) in the 9 (**I**) or 18 (**J**) months old mice. Open field test (Latency to center) in the 9 (**K**) or 18 (**L**) months old mice. The composite Z scores quantitatively demonstrate the postoperative delirium-like behavior between the control condition and anesthesia/surgery in 9 (**M**) or 18 (**N**) months old mice. The comparison of the qualitative (**O**) or quantitative (**P**) changes of the postoperative delirium-like behaviors between the 9 and 18 months old mice. The composite Z scores in the 18 months old mice were higher than those in the 9 months old mice, which demonstrates that the anesthesia/surgery may cause a greater postoperative delirium-like behaviors in the 18 months old mice than those in the 9 months old mice (**P**). N = 10 in each group. The Student’s t-test was used to analyze the data presented in (**A**–**N**). The Mann–Whitney U test was used to analyze the data presented in (**P**). * = P < 0.05.

The anesthesia/surgery differently induced behavioral changes in buried food test, Y maze test and open field test as compared to the control condition (Figure 2A–2L) between the 9 and 18 months old mice, the composite Z scores of which, obtained from the 9 and 18 months old mice, were summarized and compared (Figure 2M, 2N). Specifically, the anesthesia/surgery caused greater behavioral changes in the 18 months old mice than those in the 9 months old mice (Figure 2O), and the Student’s t-test further demonstrated that the composite Z scores in the 18 months old mice were higher than those in the 9 months old mice (Figure 2P, P = 0.019, n = 10). Note that the mice in the control group did better in some of the behavioral tests at the 9 hours after the control condition than those at the baseline, potentially due to the learning effects of these tests.

**Figure 3:**
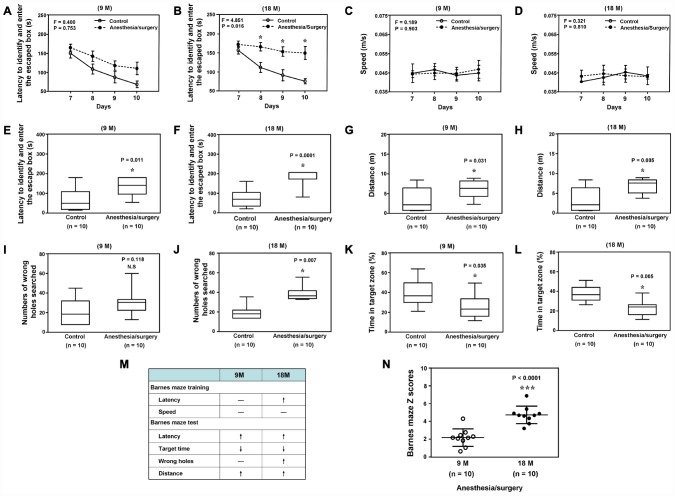
**Anesthesia/surgery induces age-dependent cognitive impairment in mice. Barnes maze training (latency to identify and enter the escape box) in the 9** (**A**) or 18 (**B**) months old mice. Barnes maze training (speed) in the 9 (**C**) or 18 (**D**) months old mice. Barnes maze testing (latency to identify and enter the escape box) in the 9 (**E**) or 18 (**F**) months old mice. Barnes maze testing (distance) in the 9 (**G**) or 18 (**H**) months old mice. Barnes maze testing (wrong holes) in the 9 (**I**) or 18 (**J**) months old mice. Barnes maze testing (time in target zone) in the 9 (**K**) or 18 (**L**) months old mice. The comparison of the qualitative (**M**) or quantitative (**N**) changes of the postoperative delirium-like behaviors between the 9 and 18 months old mice. The anesthesia/surgery causes a greater Barnes maze composite Z scores in the 18 months old mice than those in the 9 months old mice (**N**). N = 10 in each group. Two-way ANOVA with repeated measurement and post-hoc Bonferroni comparison was used to analyze the data in the (**A**–**D**). The Mann–Whitney U test was used to analyze the data presented in (**E**–**L** and **N**). * = P < 0.05.

Next, we found that the anesthesia/surgery caused significant changes in the latency to identify and enter the escape box, but not speed, as compared to the control condition in the Barnes Maze training in 18, but not 9, months old mice (Figure 3A to 3D). In the Barnes Maze testing, the anesthesia/surgery caused significant changes in the number of wrong holes searched before identifying the escaped box in 18, but not 9 month old mice. The anesthesia/surgery caused significant changes in the latency to identify and enter the escaped box, distance, and the time in target zone in both 9 and 18 months old mice (Figure 3E to 3L). However, the anesthesia/surgery caused greater behavioral changes in the Barnes maze training and testing in18 months old mice than in 9 months old mice (Figure 3M). Specifically, the anesthesia/surgery caused higher composite Z scores, representing the behavioral changes in the Barnes Maze test, in the 18 months old mice than those in the 9 months old mice (Figure 3N, P < 0.0001, n = 10, Student’s t-test). These data demonstrated that the anesthesia/surgery was able to induce age-dependent behavioral changes, e.g., postoperative delirium-like behavior and cognitive impairment, in the mice, with greater changes in the older mice.

### Anesthesia/surgery induced age-dependent changes in the gut microbiota in mice

**Figure 4:**
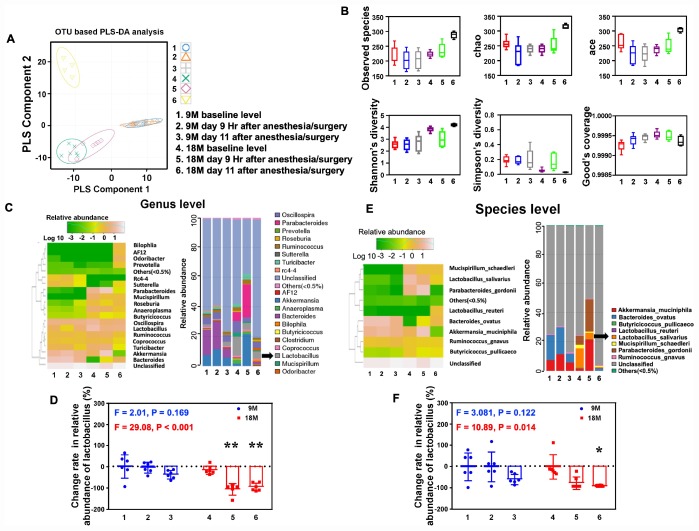
**Anesthesia/surgery induces age-dependent changes in the gut microbiota. **(**A**) OUT-based PLS-DA analysis showed that the anesthesia/surgery induced significant alterations in the component of the gut microbiota in 18, but not 9, months old mice. Different colors and shapes represented the different groups. (**B**) The number of community species between the different groups demonstrated that the anesthesia/surgery caused different microbiota diversity between the 9 and 18 months old mice, on the Observed species (top left), chao (top middle), ace (top right), Shannon’s diversity (bottom left), Simpson diversity (bottom middle), and Good’s coverage (bottom right). (**C**) Heatmap (left panel) indicated the anesthesia/surgery induced different changes in bacterial community structure represented as relative abundance shown in genus level analysis between the 9 and 18 months old mice as compared to the control condition. Anesthesia/surgery caused different profiles in the bacterial community taxonomic composition distribution (right panel) represented as relative abundance shown in genus level between the 9 and 18 months old mice as compared to the control condition. (**D**) Quantification of the change rate in the relative abundance of gut microbiota at the genus level showed the anesthesia/surgery induced significant change rate of reduction in the relative abundance of Lactobacillus as compared to the control condition at 9 hours and 11 days post-anesthesia/surgery in the 18, but not 9, months old mice (F = 2.01, P = 0.169, one-way ANOVA for the 9 months old mice, F = 29.08, P < 0.001, one-way ANOVA for the 18 months old mice). (**E**) Heatmap (left panel) indicated the anesthesia/surgery induced different changes in bacterial community structure represented as relative abundance shown in species level analysis between the 9 and 18 months old mice as compared to the control condition. Anesthesia/surgery caused different profiles in the bacterial community taxonomic composition distribution (right panel) represented as relative abundance shown in species level between the 9 and 18 months old mice as compared to the control condition. (**F**) Quantification of the change rate in the relative abundance of gut microbiota at the species level showed that the anesthesia/surgery induced significant change rate of reduction in the relative abundance of *Lactobacillus salivarius* as compared to the control condition at 9 hours and 11 days post-anesthesia/surgery in the 18, but not 9, months old mice (F = 3.081, P = 0.122, one-way ANOVA for the 9 months old mice, F = 10.89, P = 0.014, one-way ANOVA for the 18 months old mice). Partial least-squares discriminant analysis = PLS-DA, OUT = operational taxonomic unit. One-way ANOVA and post-hoc Bonferroni comparison was used to analyze the data in the (**D**–**F**). * = P < 0.05; ** = P < 0.01.

Given the findings that the anesthesia/surgery induced age-dependent changes in the behaviors in mice, next, we asked whether the anesthesia/surgery could cause age-dependent changes in gut microbiota in the mice. We found that the anesthesia/surgery induced significant alterations in the component of the gut microbiota in 18, but not 9, months old mice (Figure 4A), and different microbiota diversity between the 9 and 18 months old mice (Figure 4B). The heatmap and taxonomic composition distribution demonstrated that the anesthesia/surgery induced different change rate of reduction in the relative abundance of Lactobacillus (genus level, Figure 4C) and Lactobacillus (species level, Figure 4E). Finally, the quantification showed that the anesthesia/surgery induced a significant change rate of reduction in the relative abundance of Lactobacillus as compared to the control condition in the 18 (F = 29.08, P < 0.001 one- way ANOVA, Figure 4D, genus level; F = 10.89, P = 0.014, one-way ANOVA, Figure 4F**,** species levels), but not 9, months old mice at 9 hours and 11 days post-anesthesia/surgery. Specifically, the anesthesia/surgery induced greater change rate of reduction in relative abundance of Lactobacillus in the 18 months old mice than that in the 9 months old mice. These data demonstrated that the anesthesia/surgery was able to cause age-dependent changes in the gut microbiota.

### Anesthesia/surgery induced age-dependent changes in brain IL-6 levels, levels of synaptic marker and mitochondrial function in mice

**Figure 5:**
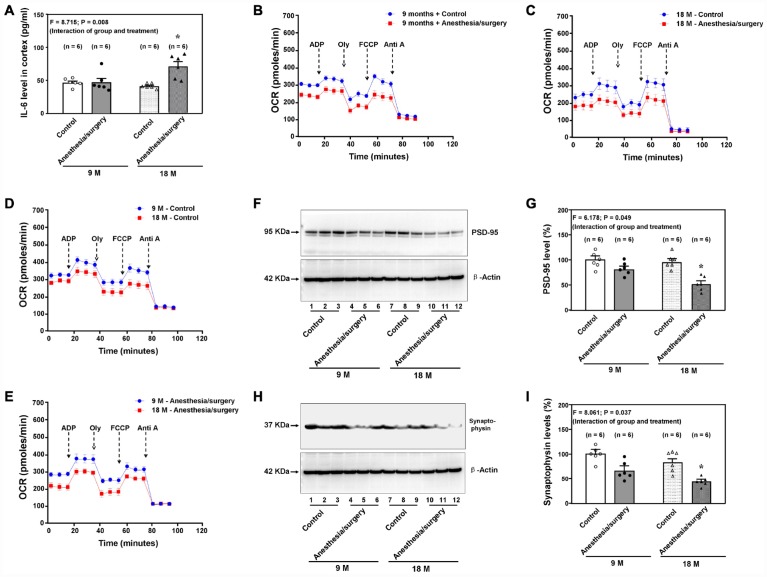
**Age-dependent postoperative changes in brain levels of IL-6, mitochondria function, and synapse levels.** (**A**). Effects of the anesthesia/surgery on the brain IL-6 levels between the 9 and 18 months old mice. The anesthesia/surgery reduces the mitochondrial function in the brain tissues of the 9 (**B**) or 18 (**C**) month old mice as compared to the control condition. The 18 months old mice have lower baseline (**D**) or greater reduction (**E**) in mitochondrial function than 9 months old mice have. (**F**) The effects of the anesthesia/surgery on PSD-95 levels between the brain tissues of the 9 months old mice and the brain tissues of the 18 months old mice. (**G**) The quantification of the Western blot showing that the anesthesia/surgery decreases hippocampus PSD-95 levels as compared to the control condition in the 18 months old mice but not in the 9 months old mice. (**H**) The effects of the anesthesia/surgery on synaptophysin levels between the brain tissues of the 9 months old mice and the brain tissues of the 18 months old mice. (**I**). The quantification of the Western blot showing that the anesthesia/surgery decreases the synaptophysin levels in the hippocampus of the 18 months old mice but not the 9 months old mice. PSD: postsynaptic density; IL-6: interleukin-6. N = 6 in each group. Two-way ANOVA and post-hoc Bonferroni comparison was used to analyze the data in the (**A**, **G** and **I**). * = P < 0.05.

Consistently, we found that the anesthesia/surgery induced age-dependent increases in brain IL-6 levels in the 9 and 18 months old mice (Figure 5A, F = 8.715, P = 0.008, two-way ANOVA, n = 6). The anesthesia/surgery decreased the brain mitochondrial function as compared to the control condition in either 9 (Figure 5B) or 18 (Figure 5C) months old mice. Moreover, the 18 months old mice had a lower baseline mitochondrial function (Figure 5D) and greater anesthesia/surgery-induced mitochondrial dysfunction (Figure 5E) than the 9 months old mice did, including base respiration rate, ADP respiration rate and maximal respiration rate (data not shown) at 9 hours after the anesthesia/surgery. In addition, the anesthesia/surgery reduced the levels of PSD-95 (Figure 5F and 5G, F = 6.178, P = 0.049, two-way ANOVA, n = 6) and synaptophysin (Figure 5H and 5I, F = 8.061, P = 0.037, two-way ANOVA, n = 6) in the hippocampus of 18 months old mice at 11 days after the anesthesia/surgery. These data demonstrated that the anesthesia/surgery caused an age-dependent increase in pro-inflammatory cytokine IL-6, mitochondrial dysfunction, and synaptic loss in the brain tissues of the mice

### Treatment with Lactobacillus rescued the anesthesia/surgery-induced behavioral and cellular changes in mice

**Figure 6:**
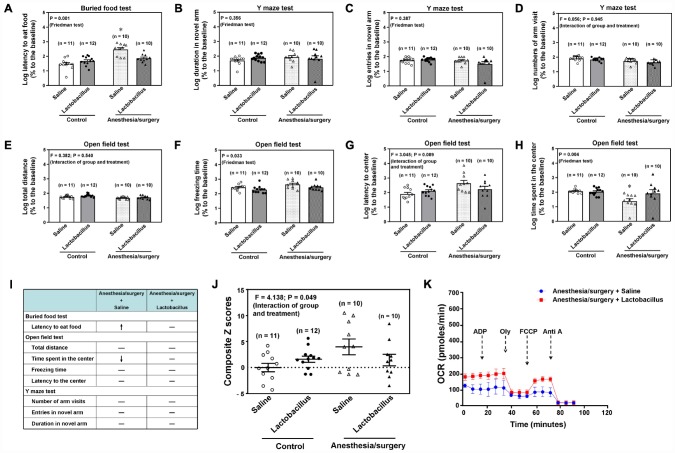
**Lactobacillus attenuate the anesthesia/surgery-induced changes in the 18 months old mice.** Interaction of anesthesia/surgery and the treatment of Lactobacillus on the buried food test (**A**), Y maze test (duration in novel arm, **B**), Y maze test (entries in novel arm, **C**), Y maze test (number of arm visit,**D**), open field test (total distance, **E**), open field test (freezing time, **F**), open field test (latency to center, **G**), open field test (Time spent in the center, **H**) in the 18 months old mice. The comparison of the qualitative (**I**) or quantitative (**J**) changes of the postoperative delirium-like behavior between the 18 months old mice received the anesthesia/surgery plus saline and the 18 months old mice received the anesthesia/surgery plus lactobacillus. The treatment of Lactobacillus attenuated the anesthesia/surgery-induced increase in the composite Z score. (**K**) The comparison of the brain mitochondrial function between the 18 months old mice received the anesthesia/surgery plus saline and the 18 months old mice received the anesthesia/surgery plus lactobacillus. The treatment of Lactobacillus attenuated the anesthesia/surgery-induced mitochondrial dysfunction. n = 10 - 12 in each group of the behavioral studies, and n = 6 in each group of the mitochondrial function studies. Friedman test was used to analyze the data in the (**A**–**C**, **F** and **H**). Two-way ANOVA and post-hoc Bonferroni comparison was used to analyze the data in the (**D**, **E**, **G** and **J**). * = P < 0.05.

To mechanistically determine the association between the anesthesia/surgery-induced changes in behaviors and gut microbiota, we assessed the effects of treatment with Lactobacillus on the anesthesia/surgery-induced changes. We found that the intragastric administration of Lactobacillus attenuated the anesthesia/surgery-induced increase in latency to eat food in the buried food test (Figure 6A, P = 0.001, Friedman’s test, n = 10 - 12), the freezing time (Figure 6F, P = 0.033, Friedman’s test, n = 10 - 12), and time spent in the center of the open field test (Figure 6H, P = 0.004, Friedman’s test, n = 10 - 12) in the 18 months old mice. Specifically, Figure 6I qualitatively demonstrated that intragastric administration of Lactobacillus attenuated the anesthesia/surgery-induced behavioral changes in the 18 months old mice. Finally, two-way ANOVA showed that there was a significant interaction of group (the control condition versus the anesthesia/surgery) and treatment (saline versus lactobacillus) on the composite Z scores (Figure 6J, F = 4.138, P = 0.049, n = 10 - 12), and the treatment with Lactobacillus attenuated the anesthesia/surgery-induced increase in the composite Z scores (Figure 6J). In addition, the treatment with Lactobacillus attenuated the anesthesia/surgery-induced mitochondrial dysfunction in the hippocampus of the 18 months old mice (Figure 6K). These data further indicated that gut microbiota played a role in the anesthesia/surgery-induced behavioral and cellular changes.

### Probiotic mitigated the anesthesia/surgery-induced behavioral and cellular changes

**Figure 7:**
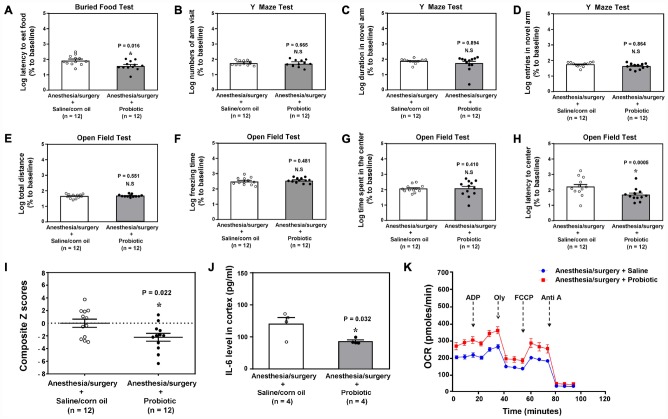
** Treatment of the postoperative delirium-like behaviors by using probiotic.**The comparison between the 18 months old mice received the anesthesia/surgery plus saline and the 18 months old mice received the anesthesia/surgery plus probiotic on buried food test (latency to eat food, **A**), Y maze test (number of arm visit, **B**), Y maze test (duration in novel arm, **C**), Y maze test (entries in novel arm, **D**), open field test (total distance, **E**), open field test (freezing time, **F**), open field test (time spent in center, **G**), and open field test (latency to center, **H**). (**I**). The comparison of the Z scores between the 18 months old mice received the anesthesia/surgery plus saline and the 18 months old mice received the anesthesia/surgery plus probiotic. There was lower composite Z score in the 18 months old mice received the anesthesia/surgery plus probiotic than that in the 18 months old mice received the anesthesia/surgery plus saline, which suggest that the treatment of probiotic mitigated the anesthesia/surgery-induced postoperative delirium-like behavior in the 18 months old mice. (**J**) The comparison of the brain IL-6 levels between the 18 months old mice received the anesthesia/surgery plus saline and the 18 months old mice received the anesthesia/surgery plus probiotic. (**K**) The comparison of the brain mitochondrial function between the 18 months old mice received the anesthesia/surgery plus saline and the 18 months old mice received the anesthesia/surgery plus probiotic. IL-6: interleukin-6. n = 12 in each group of the behavioral studies, and n = 6 in each group of the mitochondrial function studies. The Student’s t-test was used to analyze the data presented in (**A**, **B**, **F** and **J**). The Mann–Whitney U test was used to analyze the data presented in (**C**–**D** and **G**–**H**). * = P < 0.05.

Probiotic has been shown to improve cognitive function in diabetic rats [38]. We found that intragastric administration of probiotic mitigated the anesthesia/surgery-induced behavioral changes in the 18 months old mice (Figure 7A, P = 0.016, Student’s t-test, Figure 7H, P = 0.0005, Mann-Whitney test, n = 12). Specifically, Student’s t-test demonstrated that the intragastric administration of probiotic decreased the anesthesia/surgery-induced increase in the composite Z scores (Figure 7I, P = 0.022, n = 12, Student’s t-test). The treatment with intragastric administration of probiotic also reduced the anesthesia/surgery-induced increase in IL-6 levels (Figure 7J, Student’s t-test, P = 0.001, n = 4) and mitochondrial dysfunction (Figure 7K) in the hippocampus of the 18 months old mice. These data indicated that probiotic could prevent or treat the anesthesia/surgery-induced behavioral changes and the associated alterations in brain IL-6 levels and mitochondrial function.

## DISCUSSION

We determined the effects of the anesthesia/surgery on behaviors, gut microbiota, brain IL-6 levels, brain levels of synaptic marker and brain mitochondrial function in both 9 and 18 months old mice (Figure 1). We found that the anesthesia/surgery induced greater behavioral changes (Figure 2 and 3), reductions in gut Lactobacillus levels (Figure 4), increases in brain IL-6 levels, decrease in levels of synaptic marker, and brain mitochondrial dysfunction (Figure 5) in the 18 months old mice than those in the 9 months old mice. These data showed that the anesthesia/surgery induced age-dependent behavioral changes, microbiota dysbiosis, brain IL-6 increase, brain synaptic loss, and brain mitochondrial dysfunction in the mice. Moreover, the outcomes from the rescue studies with Lactobacillus (Figure 6) further demonstrated the role of gut microbiota in the anesthesia/surgery-induced changes in the behaviors and mitochondrial function. Finally, the findings that the treatment of probiotic was able to attenuate the anesthesia/surgery-induced changes (Figure 7) suggest that we may use probiotics to prevent or treat postoperative delirium-like behaviors, pending further clinical investigations.

There has been no ideal animal model to study postoperative delirium-like behaviors. Dr. Cunningham pioneered the use of the T-maze (assessing working memory) to study postoperative delirium-like behaviors in rodents [39, 40]. Delirium is characterized by several domains. The Confusion Assessment Method (CAM), a valid and widely utilized clinical instrument used to detect the presence of delirium in patients [41], consists of four clinical features: (1) acute onset and fluctuating course; (2) inattention; (3) disorganized thinking; and (4) altered level of consciousness. These features can occur naturally without learning. Therefore, we developed an animal behavioral test battery, which incorporates natural behavior (buried food and an open field test) to probe for attention and awareness as well as learned behavior (Y maze test) to assess for cognition. The tests assessed the behaviors in mice that were dependent on the presence and intactness of attention, organized thinking, and consciousness. We fully acknowledge the difficulty of modeling delirium in mice, and that no animal model of delirium can equal to human delirium. However, the battery of these behavioral tests could capture certain domains of delirium analogous to the criteria reflected in CAM, including attention, cognition, and consciousness. Thus, this model should help us to investigate the postoperative delirium neuropathogenesis. The results from the current studies using this model demonstrated that microbiota dysbiosis might contribute, at least partially, to the postoperative delirium neuropathogenesis. These findings might also suggest that aged mice, and perhaps senior patients, were more vulnerable to the development of postoperative delirium partially due to the specific changes of gut microbiota and the anesthesia/surgery-induced microbiota dysbiosis. Future studies to further test this hypothesis are warranted

Zhang et al. reported that there was different gut microbiota composition between the 8 weeks old mice with postoperative delirium-like behaviors and the 8 weeks old mice without postoperative delirium-like behaviors [32]. Specifically, the mice with postoperative delirium-like behaviors had higher levels of gammaproteobacterial, and the mice without postoperative delirium-like behaviors had higher levels of tenericutes and mollicutes [32]. Using 18 months old mice, Jiang et al. found that anesthesia/surgery was able to cause cognitive impairment in the mice and changes in gut microbiota in the mice, and moreover, the treatment of probiotics mitigated the anesthesia/surgery-induced changes in the behavior and gut microbiota in the mice [33]. Consistently, the data from the present studies demonstrated that anesthesia/surgery induced changes in mice gut microbiota and the associated changes of postoperative behaviors in the mice; and the treatment with Lactobacillus or probiotic mitigated the anesthesia/surgery-induced postoperative delirium-like behaviors in the 18 months old mice. Moreover, the present studies showed that the anesthesia/surgery was able to cause age-dependent behavioral changes, microbiota dysbiosis, increase in brain IL-6 levels, brain mitochondrial dysfunction and levels of synaptic marker in the mice

Yang et al. showed that administration of prebiotic Bimuno® [galactooligosaccharide (B-GOS) mixture] could attenuate the anesthesia/surgery-induced neuroinflammation and cognitive impairment (novel object recognition) in 8 months old rats [42]. Exercise has been shown to improve the α diversity of the gut microbiota in the low capacity runner rats, improve the β diversity in both the low and high capacity runner rats, and alter the abundance of two of the major phyla, Firmicutes and Bacteroidetes in the high capacity runner rats [43]. Zhan et al. showed that the rats with postoperative cognitive dysfunction and the rats without postoperative cognitive dysfunction had a different profile of gut microbiota [44]. Consistently, our current studies demonstrated the role of the gut microbiota in the neuropathogenesis of postoperative delirium-like behaviors. However, the current studies were different from these previous studies in the followings: (1) we compared the effects of anesthesia/surgery on the behavioral changes, gut microbiota, brain IL-6 levels, and brain mitochondrial function between 9 months old mice and 18 months old mice, and illustrated the age-dependent changes; (2) we found that anesthesia/surgery could specifically alter the gut microbiota and revealed the role of gut microbiota (e.g., lactobacillus) in the pathogenesis of postoperative delirium-like behaviors and the associated changes in brain IL-6, synaptic marker, and mitochondrial function; (3) we demonstrated that probiotic was able to mitigate the anesthesia/surgery-induced changes in behaviors and the associated increase in brain IL-6 levels and mitochondrial dysfunction. However, the limitation of the current study was that we did not assess the gut microbiota after the treatment of Lactobacillus or probiotic. The future treatment should systematically determine the interaction of anesthesia/surgery and the treatment with Lactobacillus or probiotic on the changes of gut microbiota in mice.

The anesthesia/surgery increased brain IL-6 levels in aged mice (Figure 5). These findings are consistent with the clinical observation that the elevation of plasma IL-6 level is associated with postoperative delirium in patients [45–49]. However, the results from the current studies and our previous studies [50] also showed that the increases in mouse brain and plasma levels of IL-6 were age-dependent. These findings would lead to future mechanistic investigation in animals to illustrate the underlying mechanism and clinical studies in patients to reveal the clinical relevance of the age-dependent changes in IL-6 levels. Moreover, the present studies demonstrated the age-dependent postoperative brain mitochondrial dysfunction and decreases in brain levels of PSD-95 and synaptophysin, the markers of synapse, in the mice (Figure 5). The further studies, using the established system, to determine the underling mechanism and the clinical relevance of these age-dependent changes in mitochondrial function and synapse levels are also warranted.

We only include the measurement of brain levels of IL-6, but not other pro-inflammatory cytokines, in the present studies because there are sufficient literatures suggesting the association between IL-6 and postoperative delirium in patients [45–49], and our previous studies demonstrating that the anesthesia/surgery caused age-dependent increase in blood IL-6 levels in the mice [50]. The future studies will use the established system to determine whether the anesthesia/surgery is able to induce age-dependent changes in the levels of TNF-α and IL-1β, the other pro-inflammatory cytokine, and lipopolysaccharides.

Note that there were significant variabilities and discrepancies in the data representing postoperative delirium-like behavior in the mice of the studies presented in Figure 6 and Figure 7. These variabilities and discrepancies could be due to fact that the intragastric administration could disturb the behaviors of the mice; and the non-absorb or absorb sutures received by different mice could have different effects on inflammation, leading to the different postoperative delirium-like behavior in these mice. These observed variabilities and discrepancies represent one of the limitations of the current studies. The future studies, using the established system, to illustrate the effects of intragastric administration and different kind of sutures on the postoperative delirium-like behavior are warranted.

Female mice fall into anestrus state and typically experience irregular estrous cycles around 9–12 months of age [51]. Therefore, the age-dependent changes observed in the mice in the present studies could also include the anestrus-dependent changes. The future studies should include the comparison of the observed effects between the male and female mice to rule in or rule out the potential anestrus-dependent changes in the mice.

The studies had other limitations. First, we only used female mice in the studies. Given the findings that postoperative delirium may occur more in male patients [52–54], we should employ both female and male mice in the studies. Furthermore, the observed age-dependent changes in behaviors, gut microbiota, brain IL-6 levels and mitochondrial function in the female mice could be different in male mice. However, other studies suggest that postoperative delirium may occur more in female patients [55, 56], supporting the employment of female mice in the studies to determine the mechanisms of postoperative delirium-like behavior. Nevertheless, the data from the current studies have established a system, which can be used in the future to determine whether the anesthesia/surgery can induce sex-dependent changes in delirium-like behavior, gut microbiota, brain IL-6 levels, mitochondrial function and levels of synaptic marker in mice. Second, the mice received 100% oxygen to compensate the potential hypoxia due to the isoflurane anesthesia, which could serve as a confounding factor in the studies of anesthesia on mitochondrial dysfunction since hyperoxia itself was able to cause mitochondrial stress. However, the control mice also received 100% oxygen. Moreover, blood gas studies did not show high blood PO_2_ levels. Third, there were many alterations in the gut microbiota following the anesthesia/surgery, but we only used Lactobacillus in the rescue studies. We will determine the role of other bacteria in the postoperative delirium pathogenesis in the future.

In conclusion, we have demonstrated that anesthesia/surgery induces age-dependent changes in the gut microbiota (reduction in lactobacillus), increases brain levels of IL-6, decreases levels of synaptic marker, and reduces mitochondrial function while also promoting development of postoperative delirium-like behaviors in the mice. Treatment with Lactobacillus or probiotic prior to anesthesia/surgery mitigated these detrimental effects, strongly suggesting that abnormalities in the gut microbiota contribute to the development of postoperative delirium. As such, clinical investigations of this possibility seem warranted.

## MATERIALS AND METHODS

### Mice anesthesia and surgery

The animal protocol was approved by the Massachusetts General Hospital (Boston, Massachusetts) Standing Committee on the Use of Animals in Research and Teaching (Protocol number: 2006N000219). All experiments were performed in accordance with the National Institutes of Health guidelines and regulations. Efforts were made to minimize the number of animals used. We only used female mice in the current studies because our previous studies showed that anesthesia/surgery was able to induce the postoperative delirium-like behavior [37] and postoperative cognitive impairment [57] in the female mice. Mice had 12:12 hours light:dark cycle (lights on 7:00 am) with food and water available *ad libitum*. The mice were randomly assigned to the anesthesia/surgery group or the control group by weight. Mice in the anesthesia/surgery group had a simple laparotomy under isoflurane anesthesia using the methods described in our previous studies [37, 50, 58]. Specifically, we anesthetized each of the mice using 1.4% isoflurane in 100% oxygen in a transparent acrylic chamber. Fifteen minutes after the induction, we moved the mouse out of the chamber. Isoflurane anesthesia was maintained via a cone device, and one 16-gauge needle was inserted into the cone near the nose of the mouse to monitor the concentration of isoflurane. We made a longitudinal midline incision from the xiphoid to the 0.5-centimeter proximal pubic symphysis on the skin, abdominal muscles, and peritoneum. We then sutured the incision layer by layer with 5-0 Vicryl thread. Some of the mice (e.g., the mice in the studies presented in Figure 6) received the non-absorbable suture, which could cause more inflammation; and some of the mice (e.g., the mice in the studies presented in Figure 7) received the absorbable suture, which could cause lesser inflammation. We applied EMLA cream (2.5% lidocaine and 2.5% prilocaine) to the incision site at the end of the procedure, and then every eight hours for five days or until the euthanasia of the mice, to treat the pain associated with the incision as we did in our previous studies [37, 50, 58–60]. The procedure for each mouse usually lasted about 10 minutes, and we put the mouse back into the anesthesia chamber to continue receiving the rest of the anesthesia consisting of 1.4% isoflurane in 100% oxygen for up to two hours. We used this method because surgery could potentiate the anesthesia neurotoxicity, and such a combination of anesthesia and surgery had been shown to induce postoperative cognitive impairment and postoperative delirium-like behavior [37, 50, 58, 61]. We maintained the rectal temperature of the mice at 37 ± 0.5 °C during the anesthesia/surgery by using DC Temperature Control System (FHC, Bowdoinham, Maine). We returned the mice to their home cage with food and water available *ad libitum* after recovering from the anesthesia. The mice in the control group also received 100% oxygen for two hours. The blind procedure was not possible in the studies because of the appearance of the abdominal wound in the mice.

### Treatment with Lactobacillus in mice

*Lactobacillus salivarius* (ATCC, Manassas, VA) were cultured in ATCC Medium 78 (ATCC), which consists of 20 g/L Tryptone, 5 g/L trypose, 5 g/L Yeast extract, 200 ml/L Tomato juice, 1g/L liver extract concentrate, 0.05g/L tween 80, 3g/L glucose, and 2 g/L lactose (PH 6.5, 37°C, aerobic environment). Each of the mice received 10^9^ CFU *Lactobacillus salivarius* (10^9^ CFU in 200 ul of saline) or the same volume of saline once per day for ten days by intragastric administration. Then, each of the mice waited for another ten days for the colonization of the Lactobacillus before receiving the anesthesia/surgery or the control condition.

### Treatment with probiotic in mice

The 18 months old mice were randomly divided into two groups: anesthesia/surgery plus saline/corn oil and anesthesia/surgery plus probiotic. The probiotic (Mommy’s Bliss Pharmaceuticals and Pharmapacks) contained *Lactobacillus rhamnosus* GG. The mice received 10 x 10^8^ CFU of probiotic in 100 ul or the same volume of saline/corn oil once per day by intragastric administration for 20 days before the start of the anesthesia/surgery in the mice.

### Harvest of mice brain tissues and collection of mice feces

We harvested mice hippocampus at 9 hours after the anesthesia/surgery for the ELISA studies and the measurement of mitochondrial function by using a Seahorse XFp Extracellular Flux Analyzer, and at 11 days after the anesthesia/surgery for the Western blot analysis. The mice feces were collected at one day before the anesthesia/surgery or control condition, then 9 hours and 11 days after the anesthesia/surgery or the control condition.

### DNA extraction and quantification of fecal bacteria

The relative abundance and diversity of the gut bacteria were determined by using the methods described in previous studies [62]. The 16S rRNA gene sequencing and data analysis were performed by BGI America (Cambridge, MA) using the manufacturer’s protocol. Briefly, the fecal samples were collected at the times described in Figure 1. Samples were placed in 1.5 ml tubes, snap-frozen on dry ice, and stored at −80°C until the delivery in dry ice. The 16S rRNA analysis of fecal samples was performed by amplicon-based sequencing, operational taxonomic unit (OTUs) were clustered using USEARCH (7.0.1090) with a 97% threshold. Greengene V201305 was used as a reference database for taxonomic analysis. The MixOmics package for R was used for partial least-squares discriminant analysis (PLS-DA) analysis. OTU rank curse was generated in R (V3.1.1). The tag number of each taxonomic rank (phylum, class, order, family, genus, species) or OTU in different samples were summarized in histograms generated in R (V3.1.1). In order to compare α-diversity, the OTU table was rarified, and four metrics were calculated: Chao1, Observed species, Shannon and Simpson index using MOTHUR ver.1.31.2. The β-diversity analysis was conducted as a heat map of Bray–Curtis diversity (calculated by QIIME ver.1.80) distance by using the heatmap function in the NMF package of R ver. 3.1.1. Partial least squares discrimination analysis (PLS-DA) analysis of the two principal components with the highest contribution was conducted using the mixOmics package of R ver. 3.1.1. Heatmap analysis clustering of the relative abundance of six phylogenetic levels in all samples was conducted using Euclidean and complete functions in the gplots package of R ver. 3.1.1. The change rate of relative abundance of Lactobacillus was calculated by using the raw score of the mouse received anesthesia/surgery to minus the mean of the raw scores of the mice in the control group.

### Enzyme-Linked Immunosorbent Assay (ELISA) determination of IL-6

The mouse IL-6 Immunoassay kit (Catalog number: M6000B, R&D Systems, Minneapolis, MN) was used to determine the levels of IL-6 in mouse brain tissues as described by the protocol associated with the immunoassay kit. Briefly, a monoclonal antibody specific for mouse IL-6 was coated onto the microplates. Wells were incubated for two hours at room temperature with test samples (serum) and washed for five times. Then, 100 μL of mouse IL-6 conjugate was added to each well and incubated for another two hours, and the washing was repeated twice. Finally, wells were incubated in 100 μL of substrate solution for 30 minutes and stopped with stop solution (100 μL). Determination of the optical density of each well was set at 450 nm and corrected at 570 nm as performed in our previous studies [50].

### Western blot analysis

Western blot analysis was performed using the methods described in our previous studies [63]. Hippocampus tissues were harvested from the mice at 11 days after the anesthesia/surgery or control condition. We used postsynaptic density protein (PSD)-95 antibody (1:1,000, molecular weight of 95 kDa, Cell Signaling, Danvers, MA) to detect PSD-95 level. We used anti-synaptophysin antibody (1:1,000, molecular weight of 37, Abcam, Cambridge, MA) to detect the synaptophysin levels. An β-Actin antibody (1:5,000, molecular weight of 42 kDa, Sigma, St. Louis, MO) was used to detect non-targeted protein β-Actin. β-Actin amount was used to normalize (e.g., determining the ratio of PSD-95 to β-Actin amount) protein levels and control for loading differences in the total protein amount. The quantification of the Western blot was performed as described in previous study [63]. We presented changes in protein levels in the hippocampus of the mice received the anesthesia/surgery as a percentage of those in the mice in the control group. 100% of protein level changes referred to control levels for comparison to experimental conditions. Signal intensity was analyzed using a Bio-Rad (Hercules, CA) image program.

### Measurement of mitochondrial function by using a Seahorse XFp Extracellular Flux Analyzer

Mitochondria were isolated from the hippocampus of the mice before the studies of the Seahorse XFp Extracellular Flux Analyzer. Specifically, the harvested hippocampus tissues were homogenized 20 times with a plastic tissue grinder (Thermo Fisher Scientific, Waltham, MA) in an Eppendorf tube with 600 μl of mitochondrial isolation buffer, which consisted of 70 mM sucrose, 210 mM mannitol, 5 mM HEPES, 1 mM EGTA, and 0.5% (w/v) fatty acid-free bovine serum albumin (BSA) (pH 7.2 at 37°C). The homogenate was centrifuged at 800 x g at 4 °C for 10 minutes. Then, the supernatant was centrifuged at 8,000 x g at 4 °C for 10 minutes, and the pellets were dissolved in the media without BSA, and protein concentrations were measured by bicinchoninic acid assay. The media was composed of 10 mM sodium succinate, 70 mM sucrose, 220 mM mannitol, 5 mM KH2PO4, 5 mM MgCl2, 2 mM HEPES, 1 mM EGTA, and 0.2% (w/v) fatty acid-free BSA (pH 7.2 at 37°C). The remaining supernatant was centrifuged at 8,000 x g at 4 °C for 10 minutes, and the precipitates were dissolved in the media plus BSA. After appropriate dilution with the media plus BSA, the mitochondria were seeded into an XFp cell culture miniplate (Seahorse Bioscience, Billerica, MA) at 30 μg/well. The plate was centrifuged at 2,000 x g at 4 °C for 20 minutes and incubated in the air at 37 °C for 10 minutes. A seahorse cartridge with detection probes (Seahorse Bioscience) was loaded with adenosine 5’-diphosphate (ADP, 2.5 mM), oligomycin (2 μM), carbonyl cyanide-p-trifluoromethoxyphenylhydrazone (FCCP, 4 μM), and antimycin A (3 μM) into the injection port A, B, C, and D, respectively. We then transferred the mitochondria and these reagents into the seahorse XFp extracellular flux analyzer. The real-time readings were taken for five stages: stage I, basal level; stage II, addition of ADP to measure ATP production; stage III, addition of oligomycin, a mitochondrial oxidative phosphorylation (OXPHOS) complex 5 inhibitor, to measure protein leakage; stage IV, addition of the mitochondrial OXPHOS complex 4 inhibitor carbonyl cyanide m-chlorophenyl hydrazine to measure maximal respiration; and stage V, addition of the mitochondrial OXPHOS complex 3 inhibitor antimycin A to measure nonmitochondrial respiration. Mitochondrial function was determined by measuring the oxygen consumption rate (OCR) calculated and recorded by the Seahorse XFp software.

### Behavior tests

There is no ideal animal model to study delirium. However, as we have shown previously [37], it is possible to identify a delirium-like phenotype in mice by using a battery of tests of natural and learned behaviors. We utilized that battery of tests here in the present studies. As demonstrated in the diagram (Figure 1), all of the mice had multiple behavioral tests in the order of buried food test, then open field test and finally Y maze test at 24 hours before (baseline), and then at 9 hours after the anesthesia/surgery or control condition. We performed the behavioral tests in groups of 3 mice and finished them within 50 minutes, which mimicked the specific features of clinical evaluation of delirium in patients.

### Buried food test

The buried food test was performed as described in previous studies [37, 64, 65] with modifications. Specifically, one day before the test, we gave each mouse two pieces of the sweetened cereal. On the test day, we habituated the mice for one hour before the test by placing the home cage with mice in the testing room. Then, the test cage was prepared with clean bedding (3 centimeters high). We buried one sweetened cereal pellet 0.5 centimeter below the surface of bedding so that it was not visible. The location of the food pellet was changed every time in a random fashion. We placed the mouse in the center of the test cage and measured the latency of the mouse to find and eat the food. Latency was defined as the time from when the mouse was placed in the test cage until when the mouse uncovered the food pellet and grasped it in its forepaws and/or teeth. Mice were allowed to consume the pellet they found and were then returned to their home cage. The total observation time was 5 minutes. If the mouse could not find the pellet within 5 minutes, the testing session ended, and the latency was defined as 300 seconds (5 minutes) for that mouse. We emptied the bedding from the test cage and cleaned the cage with a 70% ethanol solution after each test to prevent the transmission of olfactory cues. We also changed gloves after each test.

### Open field test

The open field test was performed as described in previous studies with modifications [37, 66, 67]. Specifically, the mouse was gently placed in the center of an open field chamber (40 × 40 × 40 centimeters) under dim light and was allowed to move freely for 5 minutes. The movement parameters of the mouse were monitored and analyzed through a video camera connected to the Any-Maze animal tracking system software (Stoelting Co., Wood Dale, IL). The total distance moved (meters), the time (seconds) spent in the center of the open field, the freezing time (seconds) and the latency to the center (the time in seconds for the mice to reach to the center at the first attempt) of the open field was recorded and analyzed. The floor of the open field was cleaned with 70% ethanol solution between each test.

### Y maze test

The Y maze test was performed as described in the previous studies with modifications [37, 68, 69]. Specifically, the Y maze, made of gray polyvinylene, was placed in a quiet and illuminated room. Each maze consisted of three arms (8 × 30 × 15 centimeters, width × length × height), with an angle of 120 degrees between each arm. The three arms included the start arm, in which the mouse starts to explore (always open); the novel arm, which is blocked at the first trial but opened at the second trial; and the other arm (always open). In the experiment, the start arm and other arm were designed randomly to avoid spatial memory error. The Y maze test consisted of 2 trials separated by a two hour inter-trial interval. The first trial (training) occurred at 7 hours after the anesthesia/surgery and was 10 minutes in duration, which allowed the mouse to explore two arms (the start arm and other arm) of the maze with the novel arm being blocked. At approximately 2 hours after the first trial, the second trial (retention) was conducted. For the second trial, the mouse was placed back in the maze in the same start arm with free access to all three arms for 5 minutes. A video camera, which was linked to the Any-Maze animal tracking system software, was installed 60 centimeters above the chamber to monitor and analyze the number of entries and the time spent in each arm. The time spent in and entries into the novel arms indicated the spatial recognition memory (learned behavior). Each of the arms of the Y maze was cleaned with 70% ethanol solution between trials.

### Calculation of composite Z-scores

The composite Z-scores to present the postoperative delirium-like behavior of each of the mice were calculated using the methods described in previous studies [37, 70] with modifications. Specifically, the Z score was calculated using the formula described by Moller et al.: Z = [ΔX Anesthesia/surgery − MEAN(ΔX) control]/SD(ΔX) control [70]. In the formula, ΔX control was the change score of mice in the control group at 9 hours after the control condition minus the score at the baseline; ΔX Anesthesia/surgery was the change score of mice in anesthesia/surgery group at 9 hours after the anesthesia/surgery minus the score at baseline; MEAN(ΔX) control was the mean of ΔX control; and SD(ΔX) control was the standard deviation of ΔX control. We also used the method for calculating a composite Z score in patients [71, 72] to determine a composite Z score for each of the mice. Specifically, the composite Z score for the mouse was calculated as the sum of the values of 6 Z scores (latency to eat food, time spent in the center, latency to the center, freezing time, entries in novel arm and duration in novel arm) normalized with the SD for that sum in the controls. Given that the reduction (rather than increase) in time spent in the center, the freezing time (open field test), the reduction in duration and entries in the novel arm (Y maze test) indicate impairment of the behavior, we multiplied the Z score values representing these behaviors by −1 prior to calculating the composite Z score using these values in the mice in either the anesthesia/surgery group or the control group.

### Barnes maze

Barnes maze test was performed by using the methods described in other studies [50, 73–76] with modifications. Barnes maze with a circular open platform (about 90 center meter diameter) was located in a quiet area. It had 20 equally spaced holes (one of these holes connects with a small dark recessed chamber called escape box) and was surrounded by a dark curtain with four simple colored-paper shapes (square, circle, triangle, and star) as markers (Stoelting, Wood Dale, IL). A video camera which could capture the entire platform was right above the platform and connected to the Any-Maze animal tracking system software (Stoelting Co., Wood Dale, IL) as described in previous studies [74, 77, 78]. The Barnes maze test in the current studies included Barnes maze training (day 7 to 10 after the anesthesia/surgery) and Barnes maze testing (11 days after the anesthesia/surgery) (Figure 1). The Barnes maze training (day 7 to day 10 after the anesthesia/surgery) consisted of 2 trials (3 minutes each trial and 15 minutes between the trials) for four days. In each trial, the mouse was placed under a bucket in the center of the circular platform for 10 seconds and was allowed to escape under the same stimulation of light and aversive noise. Once reaching the escape box, the mouse was allowed to remain in the escape box for one minute. The mouse was then removed and placed back to the home cage for 15 minutes of rest period before returning for another trial. Between each test, the Barnes maze was cleaned with 70% alcohol solution to avoid olfactory cues. After the training period, the mice had the Barnes maze testing on day 11 after the anesthesia/surgery (Figure 1). All of the mice were habituated to the maze. The mouse was placed in the escape box for two minutes and then placed directly in the hole that led to the escape box for another four minutes. Finally, the mouse was placed under a bucket in the center of the circular platform and motivated to escape under the bright light (200 Watt) and noise (85 decibels) stimulation. The mouse was gently guided to the hole connecting to the escape box when it did not go into the escape box three minutes after the light and noise stimulation. Immediately after the mouse entered the tunnel between the hole and the escape box, the buzzer was turned off. Each mouse was allowed to remain in the escape box for one minute, and then removed and placed back to the home cage. We measured and recorded the latency to identify and enter the escaped box and speed in the Barnes Maze training. In the Barnes Maze testing, we measured and recorded the latency of the mouse to identify and enter the escaped box, distance, numbers of wrong holes searched, and the time in target zone [73–76]. The decreases in escape speed would suggest impairment of locomotor activity. We used the time in the target quadrant rather than the target preference in the current studies according to the previous studies [77, 79]. The composite Z-scores of each of the mice from the Barnes Maze test were calculated also using the methods described in previous studies [37, 70] with modifications. Specifically, the Z score of the mouse to present each of the individual Barnes maze test was obtained by the following steps. First, we used the individual raw score of the behavior following the anesthesia/surgery minus the mean of the raw scores of the behavior following the control condition; second, we divided the calculated change score by SD of the raw scores of the behavior following the control condition to obtain the individual Z score of each Barnes Maze test. We added the individual Z score (total of four) to have a total Z score. Finally, we divided the total Z score of each of the mice by the SD of the total Z score of the mice in the control group to obtain the composite Z score of each of the mice.

### Statistics

The data were first examined by Shapiro-Wilk normality test. The data with normal distribution were analyzed by Student’s t-test, one-way ANOVA or two-way ANOVA, and the data with abnormal distribution were analyzed by Mann–Whitney U test or Friedman test. The data were expressed as means ± standard error of the mean (SEM) (e.g., the data of latency to identify and enter the escaped box and the speed in the Barnes Maze training) or means ± standard deviation (SD) (e.g., other data). We used 10–12 mice per group for the behavioral test, we used 6 mice per group for the Western blot, ELISA, and Seahorse XFp Extracellular Flux Analyzer studies. We chose these number of mice per group in the present studies based on the findings from our previous studies [37]. The data without normal distribution were analyzed by Mann-Whitney test, Kruskal-Wallis test or Friedman’s test, and were expressed as median and interquartile range (25% to 75%) There were significant variations in the behavioral data (some of the data were not normally distributed) following the intragastric administration of lactabocillus, probiotic and vehicle; thus, we used log transformation to transform the data (e.g., the data in Figures 6 and 7). The log transformation was performed by using Prism 8 software (Graph Pad Software, Inc, La Jolla, CA). Specifically, in the Barnes maze training, the interaction between time and group factors in a two-way ANOVA with repeated measurements was used to analyze the difference of memory curves (e.g., based on escape latency). Post hoc analysis (Bonferroni comparison) was used to compare the difference in all behavior test parameters for each testing day. Mann-Whitney test was used to compare the difference of latency to identify and enter the escaped box, distance, number of wrong holes searched, and the time in the target zone in the Barnes Maze test. Student’s t-test was used to compare the difference of other studies (e.g., the change rate of relative abundance of lactobacillus). Two way ANOVA or Friedman’s test was used to determine the interaction of treatment (e.g., control versus anesthesia/surgery) and group (vehicle versus Lactobacillus or probiotic) on the changes in the mice. *P* values less than 0.05 were considered statistically significant. Prism 8 software (Graph Pad Software, Inc) was used to analyze the data.

## Supplementary Material

Supplementary Tables
