# Supplementary material for: Anesthesia and surgery induce age-dependent changes in behaviors and microbiota
Source: Aging (Albany NY). 2020 Jan 24;12(2):1965–86. doi: 10.18632/aging.102736 (PMC7053599; doi:10.18632/aging.102736)
Supplement: Supplementary Tables [file aging-12-102736-s001..pdf]

SUPPLEMENTARY TABLES

Supplementary Table 1. The hemodynamic changes in mice after the anesthesia/surgery.

	Systolic blood pressure (mmHg)	Diastolic blood pressure (mmHg)	Mean arterial pressure (mmHg)	Heart rate (beats/minute)
		**(9 months old mice)**		
**Control**	110.60 ± 2.48	78.70 ± 3.31	89.70 ± 6.47	597.70 ± 38.11
**Anesthesia/ surgery**	104.30 ± 2.87	78.10 ± 3.52	86.27 ± 7.75	518.30 ± 22.09
**P value**	0.1488	0.7483	0.5887	0.1320
		**(18 months old mice)**		
**Control**	114.57 ± 7.75	83.27 ± 5.99	93.33 ± 8.10	602.88 ± 33.28
**Anesthesia/ surgery**	109.93 ± 7.99	75.68 ± 6.57	86.82 ± 7.89	504.68 ± 37.86
**P value**	0.5890	0.2693	0.3705	0.1955

Blood pressure and heart rate were measured in the nine months old mice in both control condition and the anesthesia/surgery condition. There were no significant differences in these measurements between the mice in the control group and the mice in the anesthesia/surgery group. SBP: systolic blood pressure; DBP: dystonic blood pressure; MAP: mean arterial pressure. N = 6 in each group.

Supplementary Table 2. The blood gas changes in mice after the anesthesia/surgery.

	PH	PCO_2 (mmHg)	PO_2 (mmHg)	Hematocrit (Hct) (%)	Na^+ (mmol/L)	K^+ (mmol/L)	Ca^{2+} (mmol/L)	HCO^{3-} (mmol/L)
				**(9 months old mice)**				
**Control**	7.30 ± 0.02	43.18 ± 3.015	103.30 ± 2.146	33.74 ± 1.595	160.0 ± 5.317	4.206 ± 0.342	1.034 ± 0.164	20.88 ± 1.787
**Anesthesia/surgery**	7.33 ± 003	42.43 ± 2.640	104.30 ± 9.280	37.30 ± 2.523	159.7 ± 2.958	4.048 ± 0.178	1.228 ± 0.087	19.00 ± 0.661
**P value**	0.2657	0.8602	0.7302	0.2187	1	0.9017	0.4127	0.2857
				**(18 months old mice)**				
**Control**	7.32 ± 0.06	40.25 ± 4.225	105.62 ± 4.462	37.80 ± 4.703	163.59 ± 2.994	4.340 ± 0.609	1.324 ± 0.043	23.69 ± 1.800
**Anesthesia/surgery**	7.34 ± 0.07	41.54 ± 4.132	102.51 ± 7.623	33.60 ± 2.995	161.63 ± 2.072	4.537 ± 0.393	1.320 ± 0.045	22.97 ± 2.523
**P value**	0.3743	0.7321	0.8651	0.0702	0.6906	0.2169	0.8960	0.4760

The blood values of pH, PCO_2, PO_2, Hct, Na+, K+, Ca^{2+}, and HCO^{3-} were determined in the nine months old mice. There were no significant differences in these measurements between the mice in the control group and the mice in the anesthesia/surgery condition. Hct: hematocrit. N = 4 - 5 in each group.
